# Long-term follow-up suggests high satisfaction rates for bulbomembranous radiation-induced urethral stenoses treated with anastomotic urethroplasty

**DOI:** 10.1007/s00345-023-04429-5

**Published:** 2023-06-14

**Authors:** John Barnard, Aron Liaw, Joel Gelman

**Affiliations:** 1 West Virginia University School of Medicine, Morgantown, WV USA; 2 University of California-Irvine, Irvine, CA USA; 3 Wayne State University, Detroit, MI USA

**Keywords:** Urethral stricture, Urethral stenosis, Radiation stricture, Radiation stenosis, Retrograde urethrogram, Prostate cancer, Radiation complications

## Abstract

**Purpose:**

To analyze patients who underwent anastomotic urethroplasty for radiationinduced bulbomembranous urethral stricture/stenosis (RIS) due to prostate cancer treatment with up to 19 years of follow-up and assess long-term patient reported outcomes (PROMs). Long-term follow-up with the inclusion of urethroplasty specific PROMs is lacking in the available research.

**Methods:**

Patients who underwent anastomotic urethroplasty for RIS were identified from 2002 to 2020. Inclusion criteria included completion of 4-month post-operative cystoscopy and PROMs including IPSS, SHIM, MSHQ-EF, 6Q-LUTS, and global satisfaction queries at 4 months. PROMs were assessed annually thereafter, and cystoscopy was performed for adverse change in PROMs or worsening uroflow/PVR parameters. PROMs were compared at pre-op, post-op, and most recent follow-up.

**Results:**

23 patients met inclusion criteria. Short-term anatomic success was 95.7%. At a mean follow-up of 73.1 months (9.1–228.9), one late recurrence occurred for an overall success of 91.3%. Significant and sustained objective improvement was identified in voiding scores, quality of life, and urethroplasty specific PROMs. Satisfaction was 91.3% despite sexual side effects, and 95.7% of patients stated they would have surgery again knowing their outcome at a mean of over 6 years’ follow up.

**Conclusions:**

RIS are challenging problems, but durable symptomatic relief is achievable in well-selected patients. Patients with bulbomembranous RIS should be appropriately counseled regarding the risk of urinary incontinence and sexual side effects after anastomotic urethroplasty. However, long-term success is high, and overall QoL will have sustained subjective improvement in most cases.

## Introduction

Radiation therapy (RT) for localized prostate cancer carries a significant risk of urethral fibrosis. Contemporary nomenclature now describes a fixed obstruction of the bladder neck, membranous or prostatic urethra as stenosis [1]. Reported rates of stenosis following RT are as high as 11% with combined external beam RT and brachytherapy [2, 3]]. The membranous urethra with varying degrees of extension into the proximal bulbar urethra is a common location for stenosis. The complications of bulbomembranous urethral reconstruction include recurrent stenosis along with de novo or worsening incontinence and ED [4, 5]. These conditions when associated with bother can result in a significant adverse impact on quality of life and patient satisfaction [6–10]. In addition, following urethroplasty for RT-induced stenosis, placement of a male sling or artificial urinary sphincter may be further complicated by both the history of RT and compromise to the anterior urethra from mobilization and transection [11–13]. Several contemporary studies, often with relatively short-term follow-up, have evaluated the short- and intermediate-term patency rates for both excisional and augmentation-based surgical approaches, and document that there is a risk of worsening erectile function and/or continence after surgery. Long-term follow-up (> 5 years) is lacking, and current literature is sparse with respect to patient satisfaction using validated questionnaires to assess voiding, sexual function, and other quality of life (QOL) measures. We hypothesized that with long-term follow-up, late recurrences may be significantly more common than previously reported given the time-dependent nature of radiation damage, chronic ischemia, and progressive fibrosis involving both the surgical bed and surrounding tissues, and that this may be reflected by declines in patient satisfaction over time. The objective of the present study was to perform an analysis of patients who underwent anastomotic urethroplasty for radiation-induced stenosis (RIS) due to prostate cancer treatment with up to 19 years of active ongoing follow-up. Emphasis was placed on patient satisfaction and QOL measured by both validated questionnaires and global patient-reported outcomes in addition to surgical success rates.

## Methods

**Fig. 1:**
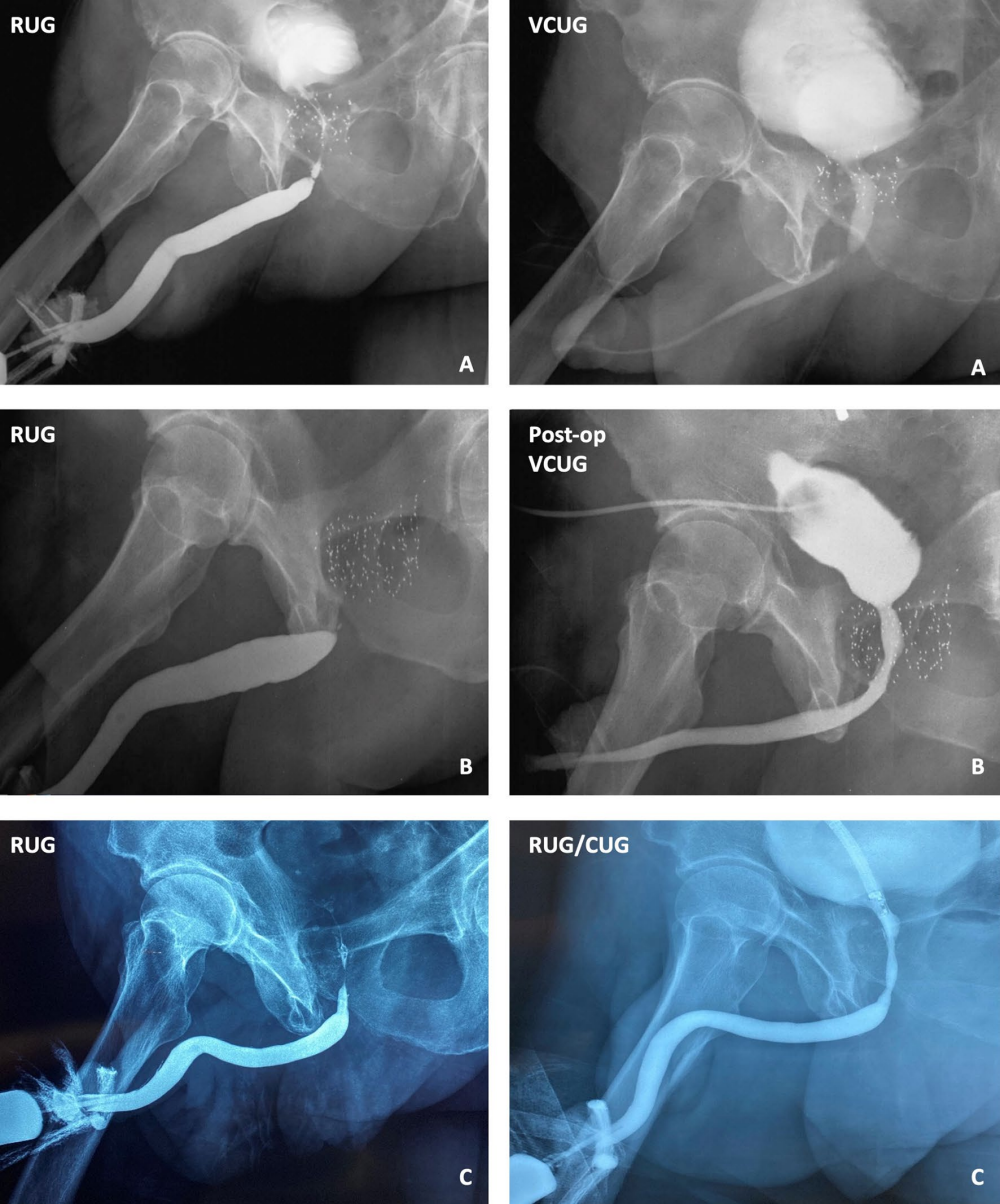
Variations in urethral stricture imaging findings include: **A** Imaging that confirmed a diagnosis of an isolated short bulbomembranous urethral stricture-stenosis amenable to urethroplasty **B** RUG in a patient in retention with a suprapubic tube who could not void volitionally. There was a total obliteration at the junction of the buIbar and membranous urethra, and the proximal extent of the stenosis could not be assessed by imaging. However, antegrade cystoscopy confirmed the proximal extent of the stenosis was distal to the verumontanum. This patient underwent anastomotic urethroplasty and the post-operative VCUG is shown. **C** RUG and combined ante- and retrograde cystourethrogram in a patient with posterior stenosis suggesting prostatic urethral involvement which was confirmed with antegrade cystoscopy. This patient was not offered urethroplasty

We performed a retrospective review of our IRB approved, prospectively maintained database for patients who underwent anastomotic urethroplasty from 2002 to 2020 for radiation-induced membranous stenosis (RIS). Our evaluation protocol included urethroscopy, a retrograde urethrogram, and a voiding cystourethrogram at the time of initial visit along with antegrade cystourethroscopy when there was a pre-existing suprapubic catheter. We identified men with RIS limited to the membranous urethra with or without extension into the proximal bulbar urethra as appropriate candidates for anastomotic urethroplasty. When there was a history of recent dilation or catheterization, a 3 month period of urethral rest was our protocol, often facilitated by suprapubic tube placement. Imaging studies generally suggested that the bladder neck was coapted at rest with a full bladder on cystogram or by antegrade cystoscopy via the suprapubic tract. If the bladder neck was not coapted at rest, patients were extensively counseled that they were high risk to not have an intact continence mechanism after posterior urethroplasty. They were also informed that this may lead to moderate to severe incontinence with associated poor quality of life. Such patients could be offered artificial urinary sphincter (AUS) placement in a staged fashion (typically at least 4–6 months post urethroplasty) and would likely be at higher risk of urethral erosion and of needing revision of the AUS. Our preference was to offer but counsel more strongly against surgery in these patients if they are tolerating the suprapubic catheter well. Surgery was offered to patients with RIS limited to the membranous or bulbomembranous urethra who had a closed bladder neck and no intraprostatic pathology such as necrosis, stones, or fistula on antegrade cystoscopy. Our protocol was to perform suprapubic catheter insertion at least 4 weeks preceding reconstruction. Many patients had a suprapubic catheter in place at initial consultation due to retention or severe LUTS; however, those with moderate LUTS were counseled regarding our preference to have a mature tract at the time of definitive intervention. On one occasion, a patient who was spontaneously voiding with only moderate LUTS despite a flow limiting stricture did not undergo suprapubic tube placement prior to urethroplasty. A pre-existing suprapubic tract facilitated the reconstruction by allowing the passage of a solid Haygrove or hollow Gelman visualizing sound to help identify the normal caliber urethra proximal to the RIS (Fig. 1). Posterior urethroplasty was performed in high lithotomy through a perineal lambda incision with splitting of the crura in all patients, infrapubectomy as deemed necessary, and excision with primary anastomosis using alternating 3–0 PDS and 3–0 Monocryl. Patients were maintained with both a suprapubic catheter and a 14 French stenting urethral catheter for 4 weeks following repair. Catheters were removed after a VCUG confirmed there was no clinically significant extravasation.

Cystourethroscopy was performed 4 months after surgery. Early anatomic technical success, a primary outcome measure, was defined as ability to pass a 16 French cystoscope with no resistance through the area of repair. Long-term success was defined as either an absence of any adverse change in voiding symptoms based on history or validated questionnaires, or confirmation of continued wide urethral patency on repeat cystoscopy if there was development of obstructive voiding symptoms, elevated residuals, or urinary tract infection. In addition, long-term outcome assessment included surveys to assess voiding [International Prostate Symptom Score (IPSS)], erectile [Sexual Health Inventory for Men (SHIM)], and ejaculatory function [4-item Men’s Sexual Health Questionnaire—Ejaculatory Function (MSHQ-EF)]. Other urethroplasty-specific questions, including a modified 6Q-LUTS, were asked in accordance with questionnaires previously published by Jackson et al. and Barbagli et al [14, 15] For patients whose initial operation was prior to 2007 and 2011 (the publication of two of the above questionnaires), only the most recent questionnaires were utilized for “long-term follow-up” for these items. Surveys utilized prior consisted of IPSS, SHIM and general questions regarding satisfaction. A two-tailed Students t-test was performed comparing pre-operative, post-operative, and current AUA symptom scores, 6Q-LUTS, and quality of life scores with significance at *p* < 0.05.

## Results

Thirty patients who underwent anastomotic urethroplasty for RIS were analyzed. Seven patients were excluded based on lack of follow-up for 4 months cystoscopy or for not providing post-operative patient-reported outcome data. Twenty-three patients met all inclusion criteria. Some patients chose not to report data within certain subsets of the post-operative survey which lead to some variations in the PROM denominators. Preoperative characteristics and intraoperative adjunctive maneuvers are included in Table 1.

**Table 1:** Preoperative patient characteristics and intraoperative adjuncts

Preoperative patient characteristics (*n* = 23)	
Age at surgery (median)	69.9 years (interquartile range ± 8.11)
Diabetes	2 (8.7%)
Smoking history	9 (39.1%)
Median time from RT to obstructive symptoms	58.83 months (range 1–165.4)
Median time from obstructive symptoms to definitive surgery	34.1 months (range 1.87–113.4)
Prior DVIU/dilation/self cath	21 (91.3%)
Median number of prior treatments	3.5 per patient
Prior transurethral resection prostate	1 (4.3%)
Preoperative suprapubic catheter	22 (95.7%)^a^
Median stenosis length based on VCUG/RUG	2 cm (IQR ± 1)
Intraoperative adjuncts	
Corporal splitting	23 (100%)
Infrapubectomy	14 (60.9%)

^a^One patient declined up-front suprapubic catheter due to moderate LUTS prior to definitive reconstruction

### Voiding outcomes

Twenty two of the twenty-three patients had wide patency when cystoscopy was performed 4 months after surgery. One patient was noted to have wide patency of the repair but a symptomatic stenosis at the bladder neck on cystoscopy at 4 months. The bladder neck was noted to have abnormal scarring but was not stenotic prior to surgery. He was treated with laser incision of the bladder neck and remains satisfied at a subsequent follow-up interval of 166 months. Although, he did not develop an anastomotic recurrence, and met our definition of cystoscopic early success as well as previously published definitions of anatomic success [8]. The patient who had a slightly less than 16 Fr caliber of the anastomosis was asymptomatic.

At the time of 4 months follow-up (and after bladder neck incision in the aforementioned patient), all patients had significant relief of urinary symptoms. Both the immediate post-op and longest follow-up AUA symptom scores were significantly lower when compared to the pre-operative AUA symptom score (Table 2). No significant difference was present between post-operative AUA symptom score and most recent AUA symptom score at mean 73.1 months follow-up. A similar trend was observed regarding the IPSS Quality of Life (QoL) score. Preoperative mean QoL score indicated patients were “Mostly Dissatisfied to Unhappy” with their urinary symptoms. Immediate post-operative mean QoL suggested patients were “Pleased to Mostly Satisfied”. Long-term QoL was no different from the immediate post-operative period and had sustained significant improvement relative to the pre-operative scores. The urethroplasty-specific modified 6Q-LUTS PROM was significantly improved in the immediate post-operative period, and the effect remained at long-term follow-up. De novo stress urinary incontinence was reported by 8 patients (34.8%). It should be noted that the pre-operative questionnaire/history did not clearly state if incontinence was present post DVIU/Dilation in those who had endoscopic management prior to surgery; therefore, this 34.8% is likely a mixed cohort with “true de novo” incontinence and “unmasked” pre-existing incontinence due to the stricture providing some resistance and acting as a continence mechanism.

**Table 2:** Patient-reported outcomes after anastomotic urethroplasty for RIS

Patient-reported outcomes (*n* = 23 unless indicated)	
Mean follow-up in months (range)	73.1 (9.1–228.9)
Recurrence at 4 months cystoscopy (%)	1 (4.3)
Late recurrence of obstructive symptoms (%)	1 (4.3)
Mean AUA symptom score	
Pre-op	19.75^a^
Immediate Post-Op (at 4 m)	11.1^b^ (ab* p = 0.0006)
Current (at Mean 73.1 m)	11.8^c^ (bc *p* = 0.4050 and ac* p = 0.0008)
Mean IPSS QoL	
Pre-op	4.5^d^
Immediate post-Op—(at 4 m)	1.7^e^ (de* p = 0.0046)
Current—(at mean 73.1 m)	2.0^f^ (ef *p* = 0.4194 and df* p = 0.0016)
Mean 6Q-LUTS score	
Pre-op	13.3^g^
Immediate post-op (at 4 m)	6.4^h^ (gh* p = 0.0023)
Current—(at mean 73.1 m)	7.3^i^ (hi *p* = 0.5000 and gi* p = 0.0090)
Erectile function after surgery	*n* (%)
No change in ED	11 (47.8)
Worsened initially but improved	2 (8.7)
Worsened and persisted	10 (43.5)
De novo stress incontinence	8 (34.8)
Post-Op SHIM score mean	4.35
Mean MSHQ-ejaculatory function (*n* = 20)	2.35
Could not ejaculate (%)	14 (70)
Ejaculatory bother (*n* = 18)	*n* (%)
None	3 (16.7)
A little	7 (38.9)
Moderate	1 (5.6)
Very	4 (22.2)
Extremely	3 (16.7)
Ejaculatory symptoms worsened post-op (*n* = 17)	*n* (%)
Yes	12 (70.6)
No	5 (29.4)
Penile sensory changes post-op	*n* (%)
Glans engorgement issues (*n* = 17)	
Yes	14 (82.4)
No	3 (17.6)
Cold glans (*n* = 15)	
Yes	7 (46.7)
No	8 (53.3%)
Penile sensory changes (*n* = 16)	
Yes	9 (56.3)
No	7 (43.7)
Overall patient satisfaction	*n* (%)
Very satisfied or satisfied	21 (91.3)
Would undergo surgery again	22 (95.7)

*Denotes statistically significant difference

Significance was defined as *P* <0.05

### Ejaculatory function outcomes

Twenty patients completed the MSHQ-EF at long-term follow-up with an average score of 2.35 with 15 being the perfect functional score. A total of 70.0% of patients reported complete inability to ejaculate. Bother scores were variable with most of the 18 patients who provided data reporting some degree of ejaculatory bother (Table 2). Twelve out of 17 (70.6%) reporting patients reported their ejaculatory function worsened post-operatively.

### Penile sensory changes

Changes to patient-reported engorgement of the glans with erections were noted in 82.4% of patients, while 17.6% reported no change post-operatively. Of the 17 reporting patients, 9 (64.2%) stated there was no swelling of the glans at all with arousal, four patients (28.6%) stated the glans became partially engorged throughout arousal, and one patient (7.1%) reported the glans became engorged initially but was not sustained. Additionally, 46.7% of our cohort reported development of a cold glans postoperatively and 56.3% endorsed a change in penile sensation involving the shaft of the penis.

### Overall patient-reported success

Our series showed 21/23 (91.3%) patients reporting long-term success as defined by a response of “Very Satisfied or Satisfied” with their overall condition. A follow-up question is asked “Would you undergo surgery again knowing your outcome” and 22/23 (95.7%) of patients stated “Yes”. Two patients met criteria for recurrence. One had a stricture of less than 16 Fr at 4 month cystoscopy which by our criteria represents technical failure; however, he was observed since asymptomatic and at most recent contact (30 months post-op) is both satisfied with his outcome and would choose to undergo surgery again knowing his outcome.

## Discussion

The AUA symptom score is one measure of functional or patient-reported success after urethroplasty; however, it does have limitations in assessing anatomic success (i.e., recurrence) following urethral reconstruction as a stand-alone tool [16–18]. Patients in our cohort had significant improvement in their urinary symptoms with a mean 9-point reduction in AUA symptom score, further quantifying the improvement in quality of life. Although the IPSS has its limitations as a urethroplasty-specific PROM as mentioned by Tam et al., the 6Q-LUTS urethroplasty-specific PROM described by Jackson et al. has improved the negative correlation between Q_max_ and patient-reported LUTS scores with a Pearson correlation coefficient of 0.75 [14, 18]. The 6Q-LUTS improves upon the IPSS for stricture disease by omitting the questions on urgency and frequency and replaces them with assessment of post-void dribbling and hesitancy which are more commonly sources of bother following urethral reconstruction [14, 16].

Unfortunately, the urethroplasty itself cannot be considered in isolation as the post-RT lower urinary tract continuously evolves and is susceptible to radiation cystitis and refractory storage LUTS at rates around 50–88% [19, 20]. Approximately 35% of the patients in this cohort reported de novo urinary incontinence after surgery which is consistent with previous studies suggesting a rate of 26–50% [6, 8]. Despite the high rate of stress incontinence, overall satisfaction with the outcome is high at 91.3%. Our preference was to counsel against urethroplasty in patients with evidence of a TUR defect or an open bladder neck at rest, particularly if they were tolerating the suprapubic catheter, because this cohort is at higher risk for severe and therefore bothersome SUI post-operatively. None of our urethroplasty cohort underwent AUS insertion, perhaps due to how strictly we selected these patients and how thoroughly we counseled against reconstruction in those deemed poorer candidates. It has been published that urethral transection is a risk factor for higher complication rates after AUS, and these complications were, therefore, avoided at the cost of some patients being resigned to suprapubic catheters. We acknowledge that this was surgeon preference, and some surgeons and patients may elect to pursue surgery anyway after an appropriate discussion of the expected outcomes and thorough informed consent. While it was not our preference, augmentation urethroplasty is another consideration to avoid transection of the urethra and theoretically some risk of cold glans, AUS complications, bulbourethral necrosis (if one or both bulbar arteries are intact post radiation and are the sole blood supply to the urethra). Anastomotic repair in the membranous urethra alone may damage the external sphincter, and when combined with RT or other damage to the bladder neck, the risk of incontinence is increased [21]. Only one patient was offered urethroplasty following prior TUR, and surgery was offered despite a known partially open bladder neck only after extensive pre-operative counseling.

Rourke et al. reported a 35% de novo ED rate post urethroplasty for RIS and other studies have suggested radiotherapy alone can result in ED in 60% of men [6, 19]. Post-operative SHIM scores were assessed in our cohort with a mean value of 4.35 with 43.5% of patients having worsened, persistent ED post-reconstruction. Ejaculatory dysfunction is another potential issue, with 70% of patients reporting anejaculation and 83.3% reporting some degree of bother. Interestingly, RT alone for prostate cancer has been associated with as high as 72% overall anejaculation rate with 16% experiencing this within 1 year of RT and 89% experiencing anejaculation by 5 years; however, a second study of 241 patients suggested 81.3% had preserved ejaculatory function on short (2.5 years) follow-up [22, 23]. To our knowledge, this is the first report of validated sexual and ejaculatory PROM data for this patient population and can add to the discussion of expectations during pre-operative counseling.

Penile sensation change following RT has sparse literature; however, a study of 190 patients by Frey et al. suggested a rate of penile sensation change of 27% with 2% reporting a cold glans and 2% reporting paresthesia of the glans or shaft [24, 25]. While this suggests that a small portion of patients can have glans or penile sensory change from RT alone, our cohort showed about half of patients will have one or both after anastomotic urethroplasty. Transection of the urethra likely is the main contributor to these changes due to alterations of neurovascular anatomy, and patients should be thoroughly counseled that this may be an outcome. It should be noted that only 1/10 (10%) of our patients that had cold glans, penile sensation change, or both expressed dissatisfaction with the outcome of their urethroplasty. A temporal relationship was noted between the onset of symptoms and time since RT, and the study only had a mean follow-up of 50 months. In our cohort, the time from RT to last follow-up PROMs after surgery has a mean of 175.8 months; therefore, the findings of the Frey et al. study are not representative of a similar patient population given the shorter follow-up interval and lack of subsequent urethral reconstruction. Our cohort endorsed a much higher rate of penile sensation change and cold glans; however, it is not possible to determine whether this is due primarily to the much longer follow-up interval and progressive radiation damage or an additive effect due to concomitant urethral reconstruction. There are no comparison studies to our knowledge that address glans engorgement, even in the post-RT setting alone, but it is noteworthy that such patients can expect some degree of decreased glans engorgement with arousal.

There are several limitations to the study. The nature of the RT, particularly dose, delivery method and setting, is not always clear. For sexual outcomes following RT, the existing literature has relatively short follow-up, so currently it is not possible to compare the urethral reconstruction cohort to matched patients who only received RT and are in some cases over 20 years post-RT treatment. This makes the delineation of what effect is due to RT alone versus any additive deleterious effect due to urethral reconstructive most challenging. It should be acknowledged that about 20% of our patients were excluded due to not meeting all inclusion criteria. Of these, only one truly never followed up for any post-op PROMs, cystoscopy, or long-term PROMs. Of the remaining patients, two had other surgeries such as concomitant augmentation, one had a salvage prostatectomy after urethroplasty but before any long-term PROM data, and the remaining patients had some combination of short-term PROM, 4 months cystoscopy, and long-term PROM data but did not have all 3. Nonetheless, the patient-reported outcome measures with validated questionnaires, penile sensory changes, and long-term follow-up represent valuable information to guide patient selection and pre-operative counseling for patients with RIS.

## Conclusions

Radiation-induced urethral stenoses are challenging problems for the reconstructive urologist, but durable relief of symptoms is achievable in well selected patients. We recognize that some authors favor substitution urethroplasty as a definitive treatment option. However, it is our experience that most RIS can be reliably treated with excision and primary anastomosis leading to a high short- and long-term success. Patients with RIS amenable to anastomotic urethroplasty should be appropriately counseled regarding the risk of post-operative urinary incontinence, ED, ejaculatory dysfunction, and penile sensory changes but can also be reassured that their overall quality of life will have sustained subjective improvement in most cases.

## Data Availability

The data that support the findings of this study are not openly available due to reasons of sensitivity and are available from the corresponding author at reasonable request. Data are located in controlled access data storage at the Center for Reconstructive Urology at University of California, Irvine.
